# Laboratory Automation in Clinical Microbiology

**DOI:** 10.3390/bioengineering5040102

**Published:** 2018-11-22

**Authors:** Irene Burckhardt

**Affiliations:** Department for Infectious Diseases, Microbiology and Hygiene, Heidelberg University Hospital, Im Neuenheimer Feld 324, 69120 Heidelberg, Germany; irene.burckhardt@med.uni-heidelberg.de; Tel.: +49-6221-56-37795; Fax: +49-6221-56-4343

**Keywords:** total lab automation, microbiology, automation microbiology

## Abstract

Laboratory automation is currently the main organizational challenge for microbiologists. Automating classic workflows is a strenuous process for the laboratory personnel and a huge and long-lasting financial investment. The investments are rewarded through increases in quality and shortened time to report. However, the benefits for an individual laboratory can only be estimated after the implementation and depending on the classic workflows currently performed. The two main components of automation are hardware and workflow. This review focusses on the workflow aspects of automation and describes some of the main developments during recent years. Additionally, it tries to define some terms which are related to automation and specifies some developments which would further improve automated systems.

## 1. What is Laboratory Automation?

“Automation is the technology by which a process or procedure is performed without human assistance” [1]. In the laboratory context this means that every switch from manual work to machines can be called automation. In this broad sense all machines in the laboratory are a kind of automation. As a consequence the introduction of centrifuges into the laboratory routine decades ago had been a form of laboratory automation already. More intuitively acceptable is the term automation for machines executing longer workflows, for example blood culture analysis with continuous growth monitoring or automated minimal inhibitory concentration (MIC) determination.

However, in the current discussion among microbiologists the terms “laboratory automation” or “total lab automation” are normally used for the automation of the diagnostic workflow including all steps from inoculation to final result. Therefore a laboratory automation system has to process different specimen containers, agar plates, broths and slides. Specimens sent to a microbiology laboratory for bacterial culture have to be inoculated and incubated. Subsequently the culture results have to be evaluated, documented and, if need be, follow-up work initiated. Not included in this form of automation is the registration of specimens at the time of their arrival at the laboratory and the generation of a report for the clinician.

Laboratory automation consists of two principal components. The first component is hardware and the second component is workflow. Adjusting existing workflows to the possibilities of the automation system is an essential step to fully tap the potential of the respective hardware. A prerequisite for these adjustments is a flexible, well-structured and adequate control system for the hardware.

Focus of this review is the total lab automation of the classic bacterial culture and the main developments and progresses during the last three to four years. Besides some remarks on the different hardware systems it emphasizes possible and/or necessary workflow alterations in the context of laboratory automation. It does not cover developments related to PCR or automation of culture of mycobacteria and fungi.

## 2. Literature Search

The Pubmed database was searched using the terms “total lab automation” and “automation microbiology”.

## 3. Definitions

### 3.1. Workflow

A workflow is a repeatable pattern of activity. A pattern consists of a sequence of operations performed by a person, a machine or a combination of both [2]. The term workflow can be used for short and for long sequences of operations. On one hand a workflow can be considered to be the basic organizational element within a laboratory. In this case the lifecycle of a patient sample in the microbiology laboratory can be described as a sequence of workflows. On the other hand, the term workflow is used to describe the whole process from arrival of a sample at the laboratory until the end of the diagnostic procedures.

### 3.2. Lean

The term lean is a technical term from manufacturing. It arose from the Toyota Production System and it describes a system to minimize “waste” during manufacturing [3]. Some authors applied the method and its principles to problems and workflows in health care [4,5]. Medicals areas where “lean” and the Toyota Production system have been discussed include pathology [6,7] clinical chemistry [8] and antibiotic stewardship [9]. The term can be adjusted to the necessities of laboratory organization. In the laboratory context waste can be unnecessary superfluous work or uneven work distribution resulting in times of excessive work or idle time.

### 3.3. Time to Result

Time to result is the time necessary to generate, produce or receive data. It is the interval between start and end of a workflow. This can be for example the time from ordering of follow-up work until reading of the result.

A lean workflow aims at the shortest possible time to result.

### 3.4. Time to Report

From the laboratory’s point of view time to report is the time between arrival of the sample in the laboratory and dispatch of the (final) report. From the clinician’s point of view time to report is the time between taking the patient sample until receipt of the report.

### 3.5. Classic System vs. Automation

The terms “classic system” and “classic workflow” are used for reference to the manual processes currently used in the majority of microbiology laboratories. In the classic system broths, slides and plates are inoculated manually, incubated in stand-alone incubators and read by looking at the actual plates. 

The terms “automation” and “automated workflow” are used for reference to processes and workflows which use machinery for inoculation, incubation and imaging and thus have a much reduced manual hands-on time per sample.

However, it is not possible to strictly distinguish between classic and automated workflows because many intermediates with different levels of automation are theoretically possible or actually exist, e.g., due to the development of automated specimen processors. Workflows with a strong emphasis on manual work fall among the definition of “classic workflow”. The term “manual workflow” is avoided on purpose because even in the automated workflow many steps have to be done manually.

### 3.6. Total Lab Automation

The terms total lab automation or total laboratory automation (TLA) are used for reference to systems which are capable of processing broths, slides and agar plates (media). These systems can inoculate, incubate and image plates.

### 3.7. What Is Quality?

To define the term quality for a microbiology laboratory the definitions from manufacturing and management can be consulted [10,11]. Quality can be described as a measure of excellence and uniformity. It can be provided by strictly following established standards to obtain results without significant variations. Quality can be implemented by defining standards and adhering to standards. In process controls are used to monitor adherence to standards.

## 4. Hardware

Looking at the available hardware one can distinguish different levels of automation. Some automates are capable of inoculation only, some systems can inoculate and incubate. These two versions can be summarized under the term “partial automation”. More elaborate systems are capable of inoculation (agar plates, broths and slides), incubation and imaging of agar plates. None of the systems can image slides or broths. These systems are the systems currently providing the maximum version of automation. Companies try to market these systems as total laboratory automation and throughout the review this term will be used to designate the most sophisticated systems. However, in fact even these systems do not provide a “total automation”. To fulfil the claim to be a “total automation” and to fulfil the definition of automation mentioned above many developments are essential (see paragraph: Wish list). Further information on the systems available can be found in two excellent reviews published in 2016 [12,13].

## 5. Workflow

The implementation of total lab automation entails auditing and review of longstanding routines. Some assays, which are easy to perform in the classic system, are cumbersome and inconvenient to perform within an automated workflow.

### 5.1. Quick Tests for Species Identification

In the classic system so called quick tests are performed directly on or from agar plates to guide follow-up work, e.g., catalase test, coagulase test, indole test, oxidase test or different latex agglutination tests. They are used to guide follow-up work, i.e., to decide whether further identification (ID) is necessary and which method is best suited for identification. Quick tests are performed directly and immediately when plates are read, that is when the technician holds the plate in her/his hand. In the automated system small tests are possible but time consuming because images are read not actual plates. To perform these tests plates would have to be called to the workstation and this would take time. In the meanwhile (until arrival of the plates) the technician would either have to wait patiently for the plate or continue reading and would have to go back to the specimen as soon as the plate is available. Both alternatives are incompatible with a lean workflow. Additionally, the time necessary to perform a quick test in an automated system is comparable to the time needed to perform a definitive ID. Therefore these tests will disappear in total lab automation and a definitive ID will be performed in all cases. Since matrix assisted laser desorption ionization – time of flight mass spectrometry MALDI-TOF MS has largely replaced biochemical identification and is still unrivaled in terms of short time to result, low cost per determination and versatility and number of identifiable species with a single workflow MALDI-TOF identification will replace the quick tests [14,15,16,17,18,19,20]. This necessary workflow change will have a collateral benefit. All bacteria will be identified to the species level. Descriptive names for bacteria like “coagulase-negative staphylococci” will disappear.

### 5.2. Quick Tests for Susceptibility Testing

Other quick tests can give a hint on susceptibility of bacteria, e.g., PBP2a (penicillin binding protein 2a) agglutination tests for a quick determination of susceptibility of staphylococci towards methicillin. These quick tests can be easily performed during classic reading but are not convenient to perform in an automated workflow for the reasons mentioned above. In the classic system these quick tests are used to determine whether further antimicrobial susceptibility testing (AST) is necessary, i.e., they are used to reduce more laborious and more expensive susceptibility tests (agar diffusion, MIC determination). However, in a certain number of cases two tests have to be performed. In an automated workflow it is easier to perform a definitive susceptibility test (agar diffusion, MIC determination) immediately and thus to avoid double testing and additional hands on time. This will increase the amount of definitive susceptibility tests. The main collateral benefit of only using definitive susceptibility tests is an increase in quality. A certain percentage of all quick test results are either false positive or false negative. These errors are avoided by the described approach.

### 5.3. Agardilution—Susceptibility Testing

Agar plates for agar dilution assays are supplemented with a defined amount of antibiotic. Because these agar dilution plates are quite expensive (compared to agar plates without supplement) laboratories tend to use one plate for testing multiple strains. This can easily be done during classic reading and follow-up work. Plates are manually labeled and depending on the skill of the technician up to eight or even more different strains can be tested on a single plate. From a quality perspective, experts have demanded for some time already that only one strain is tested per plate. There is a risk of confusion of samples and strains during follow-up work and reading of plates if multiple strains are tested per plate. However, in the laboratory routine applying multiple strains to one plate is still quite common. In the automated system testing of multiple strains on one plate is not feasible. Each plate has a unique identifier which steers the plate through the system. As soon as a plate is imaged this image is added to one sample/specimen/case in the database.

### 5.4. Effects of Laboratory Automation on Incubation Times of Agar Plates

The classic system works with incubation times measured in days. This has historical and organizational reasons. Plates are inoculated on day 0 and read once each following day until the end of the defined absolute incubation time, for example for 2 days. It is not feasible to document individual inoculation times of individual plates in the classic system. With laboratory automation plates are fully tracked as long as they stay within the system. Therefore incubation times can, and must be defined by the hour and minute. However, data on minimum and maximum incubation times are scarce. Laboratory staff normally adheres to the recommended incubation times mentioned in the package insert of the respective plate. These recommendations are normally based on data generated by the manufacturer and these data are mainly confidential and not publicly available.

From an organizational point of view two principal types of incubation time exist: (A) the incubation time after which plates are read for the first time, and (B) the incubation time after which plates are read for the last time. Choice of time of first read is dependent on the intended follow-up work. Choice of time of last read is dependent on the confidence level of a negative result. The longer the expected pathogens need for reliable growth the longer the incubation time has to be chosen. And the slowest pathogen sets the pace.

To choose time points A and B optimally we need data on growth kinetics of bacterial species. With total lab automation the generation of these kinetics is possible for the first time because plates can be imaged after individually chosen intervals of time, for example every two hours.

Looking at the results of bacterial growth kinetics three different time points can be determined: (A) first growth, that is the time point when the first bacterial mass is visible; (B) single colonies, that is the time point when the size and morphology of the colonies allow to distinguish between morphologies and allow follow-up work (species identification, susceptibility testing); and (C) typical growth, that is the time point when the morphology of the growing colonies is characteristic for a bacterial species; this time point can mainly be determined on chromogenic media and it is the color of the colony which is the decisive parameter.

#### 5.4.1. Incubation Times of Chromogenic Plates

Data on incubation times for growth of methicillin-resistant *Staphylococcus aureus* (MRSA), certain multi-drug resistant gram-negative bacteria (MDRGN) and vancomycin-resistant enterococci (VRE) on selective chromogenic plates were recently published (Burckhardt, Ann Lab Med 2019). Appearance of first growth was dependent on type and manufacturer of agar plate, bacterial species, bacterial strain and amount of bacteria inoculated. The more bacteria that were inoculated the earlier growth was detected. First growth was visible as early as four hours after inoculation. However the bacterial mass available on the agar plate at first growth was not sufficient for follow-up work. Additionally different morphologies due to bacterial mixtures could not be distinguished. Single colonies appeared later and their appearance was dependent on the same parameters as the appearance of first growth, which is agar, species, strain and inoculum. In case the plates used were not only selective but also chromogenic a third time point was defined, that is the time point of typical growth. For MRSA this was between 14 and 22 h, for VRE this was between 12 and 48 h. Moreno-Camacho and co-workers showed in their report, that growth of *Escherichia coli*, *P. aeruginosa*, *E. faecalis* and *S. aureus* on chromogenic plates was three to four hours faster in the automated system than in their classic system [21].

#### 5.4.2. Incubation Times of Agar Diffusion Plates

Incubation time for agar diffusion plates for susceptibility testing traditionally was “over night” [22] until more precise times were published by expert associations. According to EUCAST (The European Committee on Antimicrobial Susceptibility Testing) 2018 the incubation time for disk diffusion testing is 18 ± 2 h [23]. However, first data on shorter incubation times for agar diffusion were recently published. Hombach and co-workers generated growth kinetics for *Escherichia coli*, *Klebsiella pneumonia*, *Staphylococcus aureus* and *Staphylococcus epidermidis*. Their experiments with *E. coli* ATCC 25922, *S. aureus* ATCC 29213 and more than 100 clinical isolates of each species revealed, that automatically prepared disk diffusion tests can be read after 6 h for *E. coli* and *K. pneumoniae*, after 8 h for *S. aureus* and after 12 h for *S. epidermidis* [24]. The results of a study on optochin testing of pneumococci demonstrated the dependency of the result on incubation time, agar type and manufacturer. The study checked incubation times of 7, 12, 18 and 24 h and four different agar types of three different manufacturers. The authors could demonstrate that a 12 h reading time point was possible and accurate [25].

Heather and Maley used blood culture broth of bottles positive with gram-negative rods to prepare agar diffusion tests. They used disks for ampicillin, ceftriaxone, piperacillin-tazobactam, meropenem, ciprofloxacin and gentamicin. They found that growth of bacteria was sufficient for the reading of >90% of tests after 5 h of incubation. The only exception was piperacillin-tazobactam [26]. EUCAST currently addresses the matter of rapid antimicrobial susceptibility testing (RAST) directly from positive blood cultures. In the current FAQ document the method is mentioned as being “under development” [27], but official guidelines do not yet exist. However, some conference papers and a publication were presented on RAST directly from blood cultures and strains [28,29,30]. They demonstrated that meaningful reading time points were dependent on species and antibiotic. But different zone sizes had to be used compared to reading after 18 h of incubation and a new category, the category of technical uncertainty was introduced. Reading and interpretation of RAST from blood culture for *E. coli* and meropenem was already possible after 4 h. However, reading and interpretation of RAST for *E. coli* and piperacillin/tazobactam after 4, 6 or 8 h was only reliable in case of resistance. Susceptibility towards piperacillin/tazobactam could not be reliably determined after 4, 6 or 8 h because it appeared that some resistance mechanisms need longer to be expressed, i.e., the strains appeared susceptible after 4, 6 or 8 h but were resistant after 18 h of incubation. 

### 5.5. Effects of Laboratory Automation on Special Sample Types

#### 5.5.1. Effects on Blood Culture

De Socio and co-workers evaluated the effect of an automated workflow on the time to report for mono-microbial positive blood cultures [31]. In their setting the time to report was reduced by 24 h and the duration of empirical therapy (in contrast to evidence/AST-based therapy) was reduced by 32 h. The 30-day crude mortality rate was reduced by 12%. 

#### 5.5.2. Effects on Urines

Urine is a sample type which is easy to process with an automated workflow. The container for urines is simple, the sample itself is liquid, the sample can readily be acquired from the patient in sufficient amounts and for microbiological work-up only few agar plates are necessary. Additionally chromogenic media are available which ease the reading process.

As soon as urines were processed with total lab automation two effects were observed. First, the detection rate of all bacteria and especially of fastidious species rose. This effect was most pronounced for *Alloscardovia omnicolens*, *Actinotignum schaalii*, *Gardnerella vaginalis* and *Neisseria gonorrhoeae* among others [32,33].

Second, the observed times to report were reduced. Klein and co-workers automated a classic workflow where only a single read on day one was common practice in a laboratory staffed 12/7. They observed a reduced time to report of 1.5 h. Theparee and co-workers transformed their classic workflow from first read after a minimum incubation time of 14 h to reading automated images after 12 h of incubation and observed a reduction of time to report of preliminary negative results, ID and AST. The effect for preliminary negative results was most pronounced with a reduction of mean time to report of 4 h [34]. Yarbrough and co-workers [35] observed a reduction in time to report for culture negative urines of 4.5 h in a 24/7 laboratory. All three studies compared two consecutive time periods. In contrast Graham and co-workers compared the classic and automated workflow with 505 urine samples which were processed in parallel. Their data showed that results of a 14 h incubation in the automated system were “clinically concordant” to the results of a 16–24 h incubation in the classic system. However, they used a 10 µL inoculum, a bi-plate (horse blood agar/MacConkey Agar) and ambient air incubation for the automated workflow and a 1 µL inoculum, a Brilliance UTI clarity plate and ambient air incubation for the classic workflow [36].

### 5.6. Effects of Laboratory Automation on Time to Report

For the detection of MRSA a recently published study showed that the median time to report for a negative report could be reduced from 48 h to 24 h by implementation of total lab automation [37]. The study was performed such as effects of automation and extension of working/reading hours could be discerned. 

The effects on the time to report for urines and blood cultures were already described above. 

### 5.7. Effects of Laboratory Automation on Quality

Quality of work will increase if machines are used for inoculation. Machines perform their tasks in a steady and consistent fashion impossible for humans to achieve. Samples are always shaken in the same way; the streaking pattern of a machine is always identical and is neither influenced by inter- or intra-operator/technician variability. Several studies described the generation of more single colonies [21,38,39,40] which reduced the number of subcultures for necessary follow-up work (mainly for MIC determination). 

Additionally plates are imaged reliably after a defined incubation time, thus reducing variability in colony aspect and size due to differences in incubation time. Reading of plates should be more comparable and this is an important prerequisite for automated reading (see below).

The phenomenon of higher detection rates of fastidious organisms was already mentioned above. Samples which show no-growth in the classic system but show growth of pathogens in the automated system have to be considered false-negative in the classic system. Most probably the very standardized and constant incubation conditions support the growth of fastidious bacteria. Culturing, identifying and testing bacteria is the prerequisite for an accurate and evidence-based antibiotic therapy. Therefore the higher recovery rate is an improvement of quality.

### 5.8. Automated Reading

Automated reading was evaluated for chromogenic plates (MRSA [41], VRE [42]) and for urines [43,44,45]. The study on MRSA analyzed more than 50,000 plates processed at 4 different study sites. The sites worked with three different chromogenic media and images were taken immediately after manual reading, i.e., after 16–24 h after start of incubation. The authors found an overall sensitivity of 100% and an overall specificity of 90% [41]. The study on VRE analyzed more than 100,000 VRE plates at three different study sites. The sites worked with 2 different plates and images were taken after 24 h or 40 h. The authors found an overall sensitivity of 100% for VRE detection and a negative predictive value of 100% for automated reading. For reporting purposes this is extremely important because with these data at hand automated reporting of negative reports can be done without further human assistance. However, data on specificity and the positive predictive value were suboptimal with 89% and 38% respectively [42]. Glasson and co-workers evaluated automated and classical reading of almost 10,000 urines processed at three different sites and found a sensitivity of 99% and a specificity of 85% for blood and MacConkey agar. For quantification the agreement was 92% [43]. The study of Croxatto and colleagues described a sensitivity of 97% and a specificity of 94% [45]. Again the high sensitivities would allow for an automated reading and reporting.

## 6. IT

### 6.1. TLA Operating System

The requirements for an operating system are multiple. The operating system has to steer the hardware and has to enable workflows. Traceability and error management are two principal components of the operating system.

Traceability is necessary for plates, slides and broths. With automation it must be possible to follow the media through the system and to get a status information whenever possible or necessary. Nothing must get lost within the system. Ideally the system can be used for quality management as well, that is logging of lot numbers, counting of disposables like labels and consumption of media. A direct link to the purchasing department would help with organizing disposables. This is much more than any classic processing system currently provides.

Finally it is very important for the everyday management of automation that it has a user friendly and self-explaining interface in case of malfunction or problems during processing of samples. Sketches, easy to understand error messages and instructions on how to deal with errors would simplify error management.

### 6.2. Interface

The interface is the exchange point of data between operating system and LIS (laboratory information system). To enable lean workflows the interface has to be bi-directional. In general interfaces are dependent on type of LIS and operating system. Currently interfaces are custom-tailored. They have to be created and implemented before one can efficiently work with a TLA.

### 6.3. LIS

In general microbiology laboratories already work with a computerized laboratory information system. Its function is to enable administration of patient samples, documentation of arrival and processing of samples and follow-up work. It is used to create lab reports. During the implementation of a TLA the lab manager has to decide whether the LIS remains the master or becomes the slave of the TLA operating system. The decision is essential. A switch during work is not possible.

## 7. Wish List

### 7.1. Which Steps Are Still Manual?

The currently available systems automate parts of the lifecycle of a patient sample for microbiologic diagnosis. To further improve the systems, to further reduce manual work and to reach the goal of “total automation” the manual parts of the microbiological workflow have to be automated.

Two different kinds of manual work/tasks can be discerned: (A) tasks within the current automated workflow, and (B) tasks before or after or adjacent to the current automated workflow.

Tasks within the current automated workflow which are still done manually are many and varied, as follows:

(a) filling up the machine with consumables;
(b) sorting of sample containers in specialized racks;
(c) loading and unloading of racks into the specimen processor;
(d) follow-up work (that is identification, susceptibility testing, subculture);
(e) reading of plates;
(f) Generating reports for the clinicians;
(g) waste management;

Tasks before or after the currently automated workflow are many and varied, too, including:

(a) sample registration in the laboratory;
(b) incubation, assessment and documentation of broths;
(c) staining, assessment and documentation of slides;
(d) incubation and further processing of anaerobes and microaerophilic bacteria;

### 7.2. Which Data Is Needed for Optimization of Automation?

To further improve the currently available automated workflows more performance data has to be generated. An automated system inoculates and incubates differently compared to the classic system. However, the traditionally used inoculation volumes of 1 and 10 µL for first inoculation are based on the available loop sizes. Additionally all validation work for inoculation schemes for patients samples (e.g., wound swabs or respiratory samples) was done before automation was available. The current choice of media for first inoculation is solely based on the experiences with classic culture results in stand-alone incubators. The validation of agar media performance (plates and broths) was done before automation. The currently available recommendations for the length of incubation stem from the classic system. To further improve and optimize the microbiological diagnostic workflows all microbiologists are called upon to critically re-evaluate the current workflows using total lab automation. Answers to the following questions are needed to further develop, improve and optimize automation.

Are the currently used volumes of 1 and 10 µL for first inoculation sufficient for sensitivity of culture? Should we inoculate all plates with the same volume or can we increase sensitivity by using different volumes?

Which volume should be used for inoculation of broths?

Which volume should be used to prepare slides?

Which plates should be inoculated for certain patient samples?

Can other agar types improve culture results? (e.g., horse blood agar plates instead of sheep blood agar plates or tryptic soy agar based media instead of Columbia agar based media)

Can we reduce the amount of plates per sample by using different agar media?

Do we need full plates or are bi-plates comparable in terms of performance?

Is the broth really more sensitive than the agar plate? And if so, can the sensitivity of the agar plate culture be increased to match the sensitivity of the broth? Is that true for all patient samples?

What is the time to result of the gram-stained slide vs. the first ID of the agar plates? Does the result of the slide still improve antibiotic therapy? And if so for which patients and which sample type? (e.g., blood culture, wound swab, respiratory samples)

How long do we have to incubate agar plates? (e.g., first inoculation plates, subculture plates, purity controls for susceptibility testing, agar diffusion plates)

Does the introduction of an early reading (e.g., after 10 h of incubation) reduce the time to report? Does an early and preliminary report improve the outcome of the patient?

Of course this list of questions is not conclusive and during optimization of total lab automation new questions will arise.

To answer these and other questions a lot of validation work has to be done. On one hand manufacturers of agar media (plates and broths) have to provide data on the performance of the currently available products. They will have to use total lab automation to generate this data. The results should be included in the package inserts of the respective products and in the quality control certificates of the lots sold. On the other hand microbiologists are required to address these questions as well. We have to take into account that one microbiological laboratory does not work the same as another. To account for that diversity it is necessary to repeat validations. Just because a workflow works in a European laboratory does not necessarily mean it works in Asia, Australia, Africa or the Americas. Just because a new workflow improves the outcome of patients in a university hospital does not necessarily mean that it improves the outcome in an outpatient setting. Most importantly this validation data has to be published even if it is redundant. This data should not be kept internal or made confidential. These papers do not need an elaborate introduction; they do not need a lengthy discussion. They need a concise study question. They should contain detailed materials and methods sections and truthful and detailed result sections irrespective of whether the results are positive or negative. And finally they need a concise conclusion. Similar and other questions have been raised previously [46]. Because this review focuses on workflow aspects other topics like return of investment and potential reduction of personnel are not discussed here.

### 7.3. Do We Need Automated Specimen Storage?

Depending on regional guidelines patient specimens have to be stored for certain lengths of time after initial processing. The rationale behind this is contingency. In case additional investigations are necessary or in case of erroneous sample processing this can be remedied with the original specimen. The necessity for and the benefits of an automated storage system for patient specimens is dependent on the daily number of patient specimens, the necessary storage times and the number of additional investigations per day. If a future TLA version comes with an automated loading of specimens an automated storage system for the samples containers will be the logical completion of the system. For both, the automated loading and the automated storage the current versatility of sample containers is a challenge.

## 8. Discussion

Ten years ago the majority of microbiologists would have agreed that the workflows in a microbiology laboratory could not be automated in a fashion as we know automation from clinical chemistry. Apart from the complexity of samples and workflows not one microbiology laboratory works as another! However, today several systems are available which automate the workflow from incubation until reporting. In fact the available technical solutions cannot be run “without human assistance”. But the current systems have a modular structure which makes us hope that further modules will be developed and will be available soon. The first systems for automated slide interpretation [47], picking and identification of cultured bacteria [48] and automated reading are available [41,42,43,44,45]. To facilitate developments it will be important that developers secure well defined interfaces for the communication of the automated systems with other components, i.e., LIS and modules from other manufacturers. The development of locked-in systems with proprietary interfaces is understandable from marketing and economic perspectives. However, for the microbiologist who decides which system will be purchased combinability with other (preexisting) components is a very strong argument for a system and lock-in is a very strong argument against a system.

The process of total laboratory automation is a large financial investment and entails a complete reorganization of the laboratory. There is a difference between “what is possible” in terms of automation and “what is organizable” in the laboratory. Plates can be imaged at all times now, but all images have to be read. Currently samples are read once a day. Imaging after 12 and 24 h would double the reading workload. Even if the benefit for the patient warrants this new workflow someone will have to find and to pay for the personnel currently needed for this alteration. The solution to the dilemma of “what is theoretically possible” vs. “what is currently feasible” is not to refrain from automation but to intensify the efforts in developing a true automation. If algorithms can reliably read and interpret bacterial growth on agar plates restrictions in terms of imaging time points due to personnel restrictions will vanish. The main benefit for the patient would be a clinically significant reduced time to report. Even in the classic systems this can be achieved by altering workflows and techniques [49,50,51]. However, the classic system will never reach the precision and timeliness of an automated system. To max out the potential of the current total laboratory automation in terms of shortened time to report we need more data on how long incubation really has to be and which media are the best. Is a two day incubation necessary? Are 30 h enough? Do we need the broth? Is the broth really the most sensitive medium? Can we find inoculation and incubation conditions for agar plates which render the broth obsolete? 

The main pitfall during the process of automation in a laboratory and during development of additional modules is to force the automated system to do exactly what is done now in the classic system. Microbiologists should appreciate and perceive the occasion to rethink microbiology and get rid of some unnecessary longstanding and settled habits in a controlled, reflected and evidence-based manner. It is time for microbiology to advance to the 21st century.

## Abbreviations

ID	identification
AST	antimicrobial susceptibility testing
MRSA	methicillin-resistant *Staphylococcus aureus*
VRE	vancomycin-resistant enterococci
MDRGN	multi-drug resistant gram-negative bacteria
MIC	minimal inhibitory concentration
TLA	total lab(oratory) automation
MALDI-TOF MS	matrix assisted laser desorption ionization—time of flight mass spectrometry
LIS	laboratory information system
